# The Pivotal Role of Host Organizations in Enhancing Mentoring in Internal Medicine: A Scoping Review

**DOI:** 10.1177/2382120520956647

**Published:** 2020-09-30

**Authors:** Elisha Wan Ying Chia, Kuang Teck Tay, Shiwei Xiao, Yao Hao Teo, Yun Ting Ong, Min Chiam, Ying Pin Toh, Stephen Mason, Annelissa Mien Chew Chin, Lalit Kumar Radha Krishna

**Affiliations:** 1 Yong Loo Lin School of Medicine, National University of Singapore, Singapore; 2 Division of Supportive and Palliative Care, National Cancer Centre Singapore, Singapore; 3 Division of Cancer Education, National Cancer Centre Singapore, Singapore; 4 Department of Family Medicine, Yong Loo Lin School of Medicine, National University of Singapore, Singapore; 5 Star PALS, HCA Hospice Care, Singapore; 6 Palliative Care Institute Liverpool, Academic Palliative & End of Life Care Centre, University of Liverpool, Liverpool, UK; 7 Medical Library, National University of Singapore Libraries, National University of Singapore, Singapore; 8 Centre for Biomedical Ethics, National University of Singapore, Singapore; 9 Duke-NUS Graduate Medical School, Singapore; 10 PalC, The Palliative Care Centre for Excellence in Research and Education, Singapore

**Keywords:** Host organization, mentoring, medical education, mentors, mentees, continuing medical education, continuing professional development

## Abstract

In undergraduate and postgraduate medical education, mentoring offers personalized training and plays a key role in continuing medical education and the professional development of healthcare professionals. However, poor structuring of the mentoring process has been attributed to failings of the host organization and, as such, we have conducted a scoping review on the role of the host organization in mentoring programs. Guided by Levac et al’s methodological framework and a combination of thematic and content analysis, this scoping review identifies their “defining” and secondary roles. Whilst the “defining” role of the host is to set standards, nurture, and oversee the mentoring processes and relationships, the secondary roles comprise of supporting patient care and specific responsibilities toward the mentee, mentor, program, and organization itself. Critically, striking a balance between structure and flexibility within the program is important to ensure consistency in the mentoring approach whilst accounting for the changing needs and goals of the mentees and mentors.

## Introduction

Mentoring boasts many benefits. Through the provision of personalized training, learning and support, mentoring facilitates “the process by which health professionals keep updated to meet the needs of patients, the health service, and their own professional development.”^1^ Indeed, it not only enhances the academic, research, clinical, and personal development of both mentors and mentees, it also improves patient outcomes and boosts the reputation of the host organization managing the mentoring program (henceforth, the host).^2-25^ By providing opportunities for mentors and mentees to develop their social, personal, leadership, and managerial competencies,^26^ mentoring plays an integral part in the continuing medical education (CME) and continuing professional development (CPD) of physicians, nurses, and health professionals from the various allied health specialities.^27-29^

However, lapses in support and oversight of the mentor-mentee matching process, the nurturing of relationships between the mentee, mentor and the host organization, and the cultivation of a positive mentoring environment has hindered its full potential.^28-36^ With 2 recent reviews^30,31^ attributing ethical issues such as bullying and misappropriation of the mentee’s work to neglect on the part of the host, it is critical to scrutinize their role in mentoring programs.^32-41^

### Studying mentoring

A dearth of data on the role of the host in mentoring has been attributed to a number of issues.^1-20^ Perhaps most significant has been the failure of many reviews in acknowledging and contending with the impact of mentoring’s evolving, entwined, context-specific, goal-sensitive, mentee-, mentor-, relationship-, and host-dependent nature.^42-49^ This suggests that peer, near-peer, group, mosaic, network, leadership, patient, youth, family, and e-mentoring should not be mistakenly conflated nor intermixed with preceptorship, supervision, role modeling, and networking which have their own specific approach and role in education and training.^41^ Acknowledging mentoring’s context specific nature, this review will focus on the role of the host in novice mentoring which is defined as the “*dynamic, context-dependent, goal-sensitive, mutually beneficial relationship between an experienced clinician and junior clinicians and/or undergraduates focused upon advancing the development of the mentee.*”^50^ Novice mentoring is the dominant form of mentoring in medical education.^51-60^

## Methods

### Design

A systematic scoping review was adopted to identify “*the central sources and forms of evidence available*” on host organzations.^42^ The flexible nature of a scoping review allows systematic extraction, synthesis,^43^ and summarizing^44^ of actionable and applicable information across a diverse range of study formats and settings. This circumnavigates the limitations posed by mentoring’s nature^45-50^ and a paucity of articles on host organizations.^51-54^

Levac et al’s^55^ adaptations of Arksey and O’Malley’s^42^ methodological framework for conducting scoping reviews was adopted to systematically study the potential size, gaps, and scope of available literature on host organizations in novice mentoring.^56-60^ The PRISMA-P 2015 checklist was used to develop the protocol for this study.^61^

Guided by local clinicians, educators, researchers, librarians (henceforth, the expert team), and prevailing reviews of CPD practices, the 8-member research team determined the primary research question to be *“what is known about the role of the host organization in novice mentoring—particularly in Internal Medicine and its subspecialties as delineated by the American College of Physicians?”*^62^ The secondary research question was then determined to be *“what would make an effective host organization in these disciplines?”* Narrowing this scoping review’s focus on novice mentoring in Internal Medicine was largely determined by the amount of prevailing data already present in the field of mentoring in undergraduate and postgraduate medical education. These questions were designed on the PCC (population, concept, and context) elements of the inclusion criteria^63,64^ and presented in a PICOS format (Table 1).

**Table 1.** PICOs, inclusion criteria and exclusion criteria applied to database search.

PICOS	Inclusion criteria	Exclusion criteria
Population	Undergraduate and postgraduate medical students, residents, and/or postgraduate and clinical clerkship	General Surgery and Surgical Specialties
	Tutors and learners in General Medicine, including Allergy and Immunology, Clinical Medicine, Community Medicine, Dermatology, General Practice, Geriatrics, Hospital Medicine, Neurology, Palliative Medicine, and Internal Medicine (Cardiology, Endocrinology, Gastroenterology, Hematology, Immunology, Infectious Disease, Nephrology, Respiratory Medicine, and Rheumatology)	Pathology, Radiology, Pediatrics, Psychiatry, Emergency Medicine, Obstetrics and Gynecology, Anesthesia, Allied Health (Dietetics, Occupational Therapy, Psychology, Chiropractic, Midwifery, Social Work), Nursing, and Clinical and Translational Science
	Tutors and learners in Clinical, Academia or Research setting.	Non-medical professions (e.g. Science, Veterinary, Dentistry)
		Peer, Near-peer, Mosaic, and E-mentoring
		Tutoring, Preceptorship, Coaching, Role Modeling, Advising, and Sponsorship
Intervention	Interventions by HOs to create, modify, or evaluate novice mentoring processes or programs	
Comparison	Comparisons of the various characterizations, definitions, roles and descriptions of the HO and its impact upon the mentoring process, the mentoring relationship and oversight of the mentoring program	
Outcome	Concepts and constructs of HO	
	Impact of HO and its impact upon the mentoring process, the mentoring relationship, and oversight of the mentoring program	
Study design	Articles in English or translated to English	
	All study designs including	
	Mixed methods research, meta-analyses, systematic reviews, randomized controlled trials, cohort studies, case-control studies, cross-sectional studies, and descriptive papers	
	Gray Literature/electronic and print information not controlled by commercial publishing	
	Case reports and series, ideas, editorials, and perspectives	
	Year of Publication: January 2000–December 2019	
	Databases: PubMed, Embase, PsycINFO, ERIC, Cochrane Database of Systematic Reviews, Google Scholar and Scopus, GreyLit, OpenGrey, Web of Science databases	

### Sampling

A search on 7 bibliographic databases (PubMed, Embase, PsycINFO, ERIC, Cochrane Database of Systematic Reviews, Google Scholar, and Scopus) was conducted between 24th April and 12th September 2018. A search of gray literature involving GreyLit, OpenGrey, and Web of Science databases was carried out between 18th October 2018 and 17th December 2018. In order to update the search so as to include articles published up to December 2019, a subsequent search of all 10 databases was conducted between 30th December 2019 and 4th January 2020. Accounts of novice mentoring prior to the year 2000 were omitted given the propensity of these articles to neglect clear descriptions of mentoring and conflate mentoring approaches.^65,66^ The PubMed Search Strategy may be found in Supplemental Appendix A.

Upon completion of the independent searches, each member of the research team compiled a list of titles and abstracts to be reviewed. They discussed their findings online and at weekly research team meetings, achieving consensus on the final list of titles and abstracts by using Sambunjak et al’s^67^ approach to “negotiated consensual validation.”

#### Analysis

Braun and Clarke’s^68^ approach to thematic analysis was adopted in the absence of an a priori framework and a clear definition of the host in novice mentoring.

### Reiterative process

In keeping with the reiterative process outlined in Levac et al’s sixth stage, consultations with key stakeholders saw the expert team note that the themes identified were consistent with descriptions of medical education units which oversee and support multiple education programs.^69,70^ As such, drawing upon the roles of medical education units set out by the “AMEE Education Guide no. 28: The development and role of departments of medical education,”^71^ 2 independent reviewers who were not involved in the thematic analysis adopted Hsieh and Shannon’s^72^ approach to directed content analysis. This process involves “*identifying and operationalizing a priori coding categories*”^73^ which aligns with the constructivist approach adopted in this scoping review. This approach circumnavigates the wide range of research methodologies employed in the articles and prevents statistical pooling and analysis.^74-77^ Quantifying the data and tabulating the frequency by which the themes and categories emerge also aids as a proxy indicator of their significance.^78^

**Figure 1.**
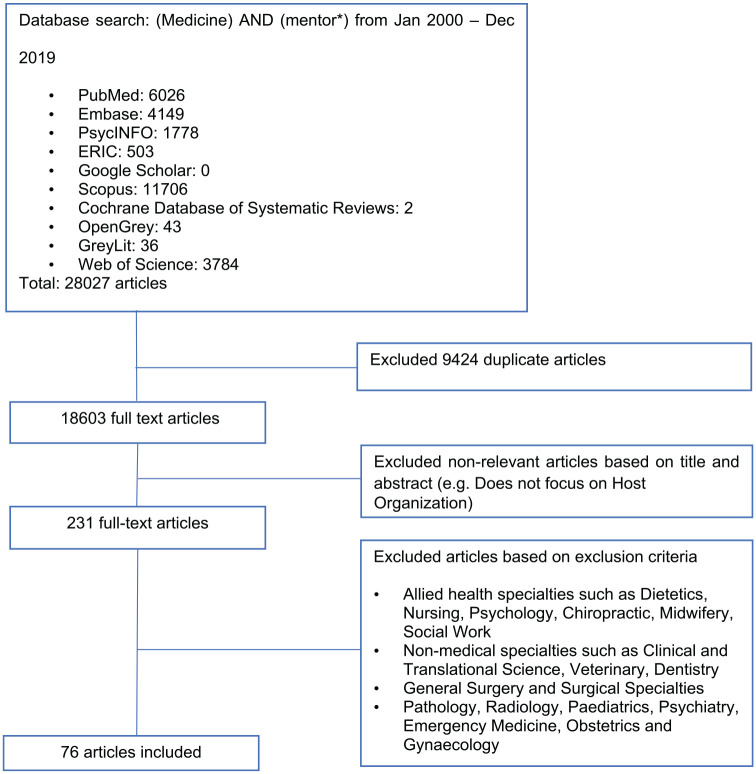
PRISMA flow chart.

In total, 18 603 abstracts were identified from the 10 databases, 231 full-text articles were reviewed, and 76 full-text articles were analyzed^79^ (Figure 1: PRISMA flow chart).

Majority of the articles surveyed mentees and mentors instead of the host organization and the articles were predominantly qualitative or quantitative, retrospective or prospective in nature. The characteristics of the 76 included articles are featured in Supplemental Appendix B.

## Quality assessment of studies

Whilst not commonly associated with systematic scoping reviews, quality assessments were deemed important to better influence and inform future practice. This sentiment was shared by the expert team. Two authors thus carried out individual appraisals using the Medical Education Research Study Quality Instrument (MERSQI)^80^ and the Consolidated Criteria for Reporting Qualitative Studies (COREQ)^81^ to evaluate the quality of the quantitative and qualitative studies included in this review.

The narrative produced was guided by the Best Evidence Medical Education (BEME) Collaboration guide^82^ and the STORIES (Structured approach to the Reporting In healthcare education of Evidence Synthesis) statement.^83^

## Results

Comparisons between the themes and categories identified using thematic and directed content analysis revealed significant consistencies.

(1) “Defining” and secondary roles

An overwhelming majority of the included articles defined the host organization by the roles they play in their respective mentoring programs.^67,83-141^ These papers suggest that the “defining” roles^47^ of the host include:

- establishing and/or complying with overarching goals, clinical standards, and curriculum requirements^84,86-92,140^
- designing,^83,88,92,95-102,132,135^ influencing,^47,90,92-94,122^ and overseeing the mentoring program^88,95,98,102-107^
- and nurturing the mentoring culture^84,85,90-92,97,101,106,108-110^ and mentoring relationships^83,85,88,92,95-102^

Characteristics of an *effective* host, in particular, were determined to be their ability to:

- provide consistent leadership^47,67,84,91,93,96,102,107,137,142-144^
- proactively support mentor and mentee participation^47,67,83,85,93,96,101,103,107,111,118,120,129,134,142-145^
- cogently facilitate all mentoring processes^47,67,87,91,92,99,101,106,107,111,118,127,138,143,144,146^
- proactively gather and revert feedback on the mentoring program and the mentoring relationships within^47,89,101,116,126,134,142,146-148^
- and, finally, successfully initiate curricular reform to better meet the needs of their participants^107,137,144,146^

In addition, the secondary roles of the host comprise of supporting patient care and specific responsibilities toward the mentee, mentor, the overall program, and the organization itself. These are outlined in Table 2.

**Table 2.** Secondary Roles of Host Organization.

Roles in	Elaboration	References
Patient Care	Supports patient care delivery, safety, and health outcomes	^92,110,124,125^
Mentee and Mentors	Supports their personal	^47,85,88,92,108,113,120,142,144,149,150^
	Professional and career development	^47,83,85,88,91,92,98,106,108,112,113,116-120,126,129,137,142-144,147,149-151^
	And addresses heavy workloads, stress, and anxiety amongst mentors and mentees	^88^
Program	Reduces the proportion of ineffective matches and unnecessary evaluations of mentors, mentees, and the mentoring process to sustain the viability of the program	^47,88,126,129^
Organization	Maintains:	
	Organizational practice and collaborations	^47,83,85,92,124,144^
	Research development	^47,92,108,111,128,133,142-144,147^
	Faculty development	^83,85,88,92,108,116,128,129,138,142,143,146^
	Satisfaction and retention	^83,88,92,96,108,110,116,126,129,138,144,146,152,153^
	Sustainability and productivity of the program	^47,85,96,118,124,142-144,153^

(2) Balancing structure and flexibility

Although not a defining characteristic of the host organization, a key finding which emerged in numerous papers was the need for the host to balance structure and flexibility within the program.

Establishing a mentoring structure serves to ensure fairness and consistency in the mentoring approach and experience for all.^67,83,85,88,90-93,95-101,104,107,111,118,121,123-125,129,133,134,136,138,142,144^ Rigorous oversight and the just provision of support, in turn, influences the mentoring culture.^47,92,104,106,107,142,151^ It ensures transparent communication of the program’s mentoring philosophy, mentoring approach, as well as the goals and values of the program.^47,88,95,98,101,103,104,124,149^ It also facilitates recruitment and retention of mentors through the provision of financial remuneration,^84,86,88,93,97,102,104,124,129,130,134,139,142,144,150^ opportunities for academic promotion,^83,86,93,106,138,139^ formal recognition of their time and efforts,^25,83,84,86,90,92,96,97,124,134,138,139,144,150^ timely access to facilities, resources,^47,105,144^ research funding,^84,136,154^ and protected time.^25,67,83,84,90,92,95-97,133,134,139,142,144,154^

Yet, the host must also allow for flexibility^47^ within the mentoring program so as to adapt to the evolving mentee-, mentor-, host organization-, and relationship-dependent nature of each individual mentoring relationship.^114^ This is critical in enhancing the mentee’s sense of autonomy, connectivity, and advocacy.^114,144^ It is of note that flexibility is also encapsulated within the clinical standards and codes of conduct set out by prevailing host organizations.^47,84,86-92^ As outlined in Table 3, structure and flexibility within the mentoring program is evident in the various stages of the mentoring process.

**Table 3.** Structure and Flexibility in Stages of Mentoring Processes.

Stage	Elaboration	References
Structure		
Matching Stage	The host establishes its selection, vetting, matching and training for mentees and mentors	^47,67,83,85,88,90-93,95-101,104,107,111,118,121,123-125,129,133,134,136,138,142^
	And may use contractual agreements	^67,90,92-94,101,102,131^
	To align expectations	^47,84,85,88,90,92,97,101,108-110,131,139,142,144^
	And clarify goals, timelines, and roles	^53,106,112,115,118,119,138,156^
	And responsibilities	^53,54,106,112,115,118,119^
Pre-mentoring Stage	Sets out its own objectives establishes and oversees the entry criteria, goals, selection, and matching processes	^84,92,97,99,101,103,105,124,142,144,147^
	And the mentoring approach	^85,88,95,98,101,103,104,142^
	Within a formal curriculum	^47,87,88,94,95,98,104,111,116,117,119,126,130,134,136,139,148,150,155,156^
^Flexibility^		
Matching Stage	Accounts for the mentor’s and mentee’s goals and interests	^47,52,84,85,92,97,101,111,126,132,142,144^
	Personalities	^101^
	Preferences on how they would like to initiate mentoring relationships	^67,101,110,111,129,132^
	Gender	^83,96,132^
	Background	^87,96^
	Ethnicity	^67,92^
Pre-mentoring stage	Flexibility is apparent in the:	^47,67,90,92,97,101,106,108,109,117,129^
	structure, form and frequency of meetings, codes of conduct, roles and responsibilities and standards of practice established	^47,87,90,92,96,97,101,141^
	consideration of individual mentee’s and mentor’s expectations	^47,108,111^
	goals	^47,85,105,106,147^
Mentoring Process	Adaptable	^25,47,87,90,92,96,97,101,141^
	And longitudinal evaluations are employed to account for changes in the mentoring relationships and shifts in individual academic, social, research, and personal situations of mentees and mentors	^84-87,89-93,96,97,102,115,142,144,149^
	Flexibility is also evident as hosts respond and adapt its approach and support in response to appraisals	^67,87,91,92,99,101,106,107,111,118,127,138,146^

## Discussion

In addressing its primary and secondary questions, this SSR characterizes the host organization as a “team of educators and administrators^83,102,104,112,116^ with common values, goals and views on education and clearly delineated roles and responsibilities ^86,87,90-92^ who collaborate through coordinated lines of communication^104^, assessment, and reporting ^88,95,98,103,104^ in order to realize their “defining” and secondary roles. The “defining” roles of the host include establishing, nurturing, and overseeing mentoring relationships whilst ideally offering both structure and flexibility within the mentoring program.”^83,84,86-110^

To realize their “defining” roles, the host should design and incorporate the mentoring program as part of the formal curriculum.^87,88,94,95,98,104,111,116,117,119,126,130,134,136,139,148,150,155^ This will provide the program leaders with administrative, financial, and training support that will help streamline their response to the changing needs of the stakeholders and the mentoring process.^67,87,91,92,99,101,106,107,111,118,127,138,146^ Such resources will ensure that codes of conduct, standards of practice, timelines, roles, and responsibilities to be adhered to by mentees, mentors and the educationalists and administrators designing and spearheading the program are clearly overseen.^84,86-92^ A consistent framework is also critical in ensuring that mentoring takes place within reasonable boundaries.^30,31^ Establishing an implicit or contractual^67,90,92-94,101,102,131^ agreement between mentees and mentors on the expectations of the mentoring program would minimize the risk of misdemeanors and breaches in ethical conduct.

To ensure a flexible approach, the host must adopt adaptable, context sensitive, and stakeholder-specific assessment methods to provide mentees, mentors, and the mentoring relationship with personalized, appropriate, specific, timely, holistic, accessible, and longitudinal support.^25,67,84,86,87,89-93,96,97,99,101,102,106,107,111,115,118,127,138,141,146,149^ Adaptations should be guided by consistent evaluations of the mentee’s, mentor’s, and the host organization’s changing needs and goals. In the absence of a specific assessment tool, a combination of tools and assessors may be considered.^83,88,91,92,96,98,106,108,110-113,116-120,124-126,128,129,133,137,138,146,147,149-153^

The host should also work to establish an accessible and robust platform for mentors and mentees to communicate freely and confidentially. Such a platform would also encourage mentors and mentees to attune themselves and respond to any changes during the course of their mentoring relationship. To facilitate this, they should be provided with pre-mentoring workshops and longitudinal training programs to develop their communication and online literacy skills. Such skills will help them to circumnavigate obstacles such as conflicting schedules amidst urgent deadlines that may impede the progress of their mentoring projects.

Overall, these considerations will provide the host with the opportunity to deliver consistent, timely, appropriate, longitudinal leadership^47,67,84,91,93,96,102,107,137,142-144^, holistic support for the matching,^47,67,83,85,93,96,101,103,107,111,118,120,129,134,142-145^ and mentoring process^47,67,87,91,92,99,101,106,107,111,118,127,138,143,144,146^, personalized, specific and comprehensive feedback to all participants ^47,89,101,116,126,134,142,146-148^ and the successful development and execution of crucial curricular reform.^107,137,144,146^

## Limitations

Too narrow a picture of the host organization may have been sketched in this scoping review given that it was explored in isolation from factors such as the dynamic nature of mentoring relationships, structures, environments, and even mentee-mentor matching processes. Concurrently, given the context-specific nature of the host and their roles in mentoring programs, conflation within the included articles of different healthcare, educational and CPD systems across different national and international contexts may prove to be problematic.

These limitations are compounded by the scoping review’s focus on articles published or translated into English. As a result, much of the data comes from North America and Europe, potentially skewing perspectives and raising questions as to the applicability of these findings in other healthcare settings. In addition, despite using the Endnote software to carry out independent searches and consolidation of the findings, relevant articles may have been unintentionally omitted.

However, despite these limitations, this scoping review was carried out with the required rigor and transparency advocated by Arksey and O’Malley,^42^ Levac et al,^55^ and Pham et al.^157^ As a result, we believe that the findings will help to inform the design and oversight of future mentoring programs. We also believe that this review may be of interest to educators and program designers in settings beyond the mentoring landscape due to the potential applicability of the findings to other aspects of medical education.

## Directions for future research

This scoping review evidences the critical role of the host in mentoring programs and hints at their applicability to undergraduate and postgraduate medical education. Riding on ever improving communication technology and advances in the dissemination of information, the increasing use of technology-enhanced mentoring platforms will also see ever increasing demands for transparency and accountability. There is a need to conduct closer evaluations of all intra and interprofessional mentoring interactions to ensure that personal and professional boundaries are maintained with codes of conduct and standards of practice strictly adhered to.

Prospective studies should be conducted to better understand how balance between structure and flexibility can be better struck to ensure maximum efficacy. However, it is only with the curation and validation of effective assessment tools accounting for mentoring’s evolving nature that mentoring can realize its true potential in CPD programs.

## Lessons for practice

(a) Mentoring’s role in CPD hinges on effective support and oversight by the host organization. This may be facilitated through the development of a formal mentoring program that is overseen by the wider education and administrative team.
(b) Collaborative efforts between educators and administrators are required to ensure that a clear organizational structure is established with the aim of meeting the critical “defining” roles of the host. These comprise of establishing, nurturing, and overseeing the mentoring relationships whilst balancing structure and flexibility within the program. This process must be guided by clear outcome measures, codes of conduct, standards of practice, and assessment points.
(c) Members of the host must be trained, briefed, supported, and appraised throughout the mentoring program. Their roles, responsibilities and lines of reporting should be clearly established.
(d) Mentoring in CPD should be run as a longitudinal program that is in turn supported by mentee and mentor training workshops.
(e) Mentoring’s role in CPD is to facilitate personalized social, academic, and leadership development opportunities especially when used in conjunction with e-mentoring. However, the effectiveness of such an approach pivots upon the host’s ability to assess and respond to the evolving needs of the mentee, mentor and the mentoring relationship.

## Supplemental Material

Supplementary_material – Supplemental material for The Pivotal Role of Host Organizations in Enhancing Mentoring in Internal Medicine: A Scoping Review Click here for additional data file. Supplemental material, Supplementary_material for The Pivotal Role of Host Organizations in Enhancing Mentoring in Internal Medicine: A Scoping Review by Elisha Wan Ying Chia, Kuang Teck Tay, Shiwei Xiao, Yao Hao Teo, Yun Ting Ong, Min Chiam, Ying Pin Toh, Stephen Mason, Annelissa Mien Chew Chin and Lalit Kumar Radha Krishna in Journal of Medical Education and Curricular Development

## References

[1] Peck C McCall M McLaren B Rotem T. Continuing medical education and continuing professional development: international comparisons. BMJ. 2000;320:432-435. 10669451 10.1136/bmj.320.7232.432 PMC1117549

[2] Alleyne SD Horner MS Walter G Fleisher SH Arzubi E Martin A. Mentors’ perspectives on group mentorship: a descriptive study of two programs in child and adolescent psychiatry. Acad Psychiatry. 2009;33:377-382. 19828850 10.1176/appi.ap.33.5.377

[3] Andre C Deerin J Leykum L. Students helping students: vertical peer mentoring to enhance the medical school experience. BMC Res Notes. 2017;10:176. 28464902 10.1186/s13104-017-2498-8 PMC5414204

[4] Buddeberg-Fischer B Vetsch E Mattanza G. Career support in medicine—experiences with a mentoring program for junior physicians at a university hospital. Psychosoc Med. 2004;1:Doc04. 19742055 PMC2736485

[5] Bussey-Jones J Bernstein L Higgins S, et al Repaving the road to academic success: the IMeRGE approach to peer mentoring. Acad Med. 2006;81:674-679. 16799297 10.1097/01.ACM.0000232425.27041.88

[6] Chen MM Sandborg CI Hudgins L Sanford R Bachrach LK. A multifaceted mentoring program for junior faculty in academic pediatrics. Teach Learn Med. 2016;28:320-328. 27054562 10.1080/10401334.2016.1153476 PMC5003054

[7] Files JA Blair JE Mayer AP Ko MG. Facilitated peer mentorship: a pilot program for academic advancement of female medical faculty. J Womens Health (Larchmt). 2008;17:1009-1015. 18681821 10.1089/jwh.2007.0647

[8] Fleming GM Simmons JH Xu M, et al A facilitated peer mentoring program for junior faculty to promote professional development and peer networking. Acad Med. 2015;90:819-826. 25830537 10.1097/ACM.0000000000000705 PMC4446138

[9] Kashiwagi DT Varkey P Cook DA. Mentoring programs for physicians in academic medicine: a systematic review. Acad Med. 2013;88:1029-1037. 23702518 10.1097/ACM.0b013e318294f368

[10] Lewellen-Williams C Johnson VA Deloney LA Thomas BR Goyol A Henry-Tillman R. The POD: a new model for mentoring underrepresented minority faculty. Acad Med. 2006;81:275-279. 16501276 10.1097/00001888-200603000-00020

[11] Lord JA Mourtzanos E McLaren K Murray SB Kimmel RJ Cowley DS. A peer mentoring group for junior clinician educators: four years’ experience. Acad Med. 2012;87:378-383. 22373635 10.1097/ACM.0b013e3182441615

[12] Pololi LH Evans AT. Group peer mentoring: an answer to the faculty mentoring problem? A successful program at a large Academic Department of Medicine. J Contin Educ Health Prof. 2015;35:192-200. 26378425 10.1002/chp.21296

[13] Pololi LH Knight SM Dennis K Frankel RM. Helping medical school faculty realize their dreams: an innovative, collaborative mentoring program. Acad Med. 2002;77:377-384. 12010691 10.1097/00001888-200205000-00005

[14] Singh S Singh N Dhaliwal U. Near-peer mentoring to complement faculty mentoring of first-year medical students in India. J Educ Eval Health Prof. 2014;11:12. 24980428 10.3352/jeehp.2014.11.12 PMC4309947

[15] Welch JL Jimenez HL Walthall J Allen SE. The women in emergency medicine mentoring program: an innovative approach to mentoring. J Grad Med Educ. 2012;4:362-366. 23997883 10.4300/JGME-D-11-00267.1 PMC3444192

[16] Toh YP Lam BL Soo J Chua KLL Krishna L. Developing palliative care physicians through mentoring relationships. Palliat Med Care. 2017;4:1-6.

[17] Yeam C Loo WT Ee MH Kanesvaran R Krishna L. An evidence-based evaluation of prevailing learning theories on mentoring in palliative medicine. Palliat Med Care. 2016;3:1-7.

[18] Wu J Wahab MT Ikbal MFBM Loo TWW Kanesvaran R Krishna LKR Toward an interprofessional mentoring program in palliative care—a review of undergraduate and postgraduate mentoring in medicine, nursing, surgery and social work. J Palliat Med. 2016;6:1-11.

[19] Wahab MT Ikbal MFBM Wu J Loo WTW Kanesvaran R Krishna LKR Creating effective interprofessional mentoring relationships in palliative care: lessons from medicine, nursing, surgery and social work. J Palliat Med. 2016;6: 1-10.

[20] Loo WTW Ikbal MFBM Wu JT, et al Towards a practice guided evidence based theory of mentoring in palliative care. J Palliat Care Med. 2017;7:296.

[21] Tan B Toh YL Toh YP Kanesvaran R Krishna LKR Extending mentoring in palliative medicine-systematic review on peer, near-peer and group mentoring in general medicine. J Palliat Med. 2017;7:323.

[22] Kalen S Ponzer S Seeberger A Kiessling A Silen C. Longitudinal mentorship to support the development of medical students’ future professional role: a qualitative study. BMC Med Educ. 2015;15:97. 26037407 10.1186/s12909-015-0383-5 PMC4458053

[23] Balmer D D’Alessandro D Risko W Gusic ME. How mentoring relationships evolve: a longitudinal study of academic pediatricians in a physician educator faculty development program. J Contin Educ Health Prof. 2011;31:81-86. 21671273 10.1002/chp.20110

[24] Rashid P Narra M Woo H. Mentoring in surgical training. ANZ J Surg. 2015;85:225-229. 25649003 10.1111/ans.13004

[25] Jackson VA Palepu A Szalacha L Caswell C Carr PL Inui T. “Having the right chemistry”: a qualitative study of mentoring in academic medicine. Acad Med. 2003;78:328-334. 12634219 10.1097/00001888-200303000-00020

[26] Campbell C Silver I Sherbino J Cate OT Holmboe ES. Competency-based continuing professional development. Med Teach. 2010;32:657-662. 20662577 10.3109/0142159X.2010.500708

[27] Lin J Chew YR Toh YP Krishna LKR Mentoring in nursing: an integrative review of commentaries, editorials, and perspectives papers. Nurse Educ. 2018;43:E1-E5. 10.1097/NNE.0000000000000389 28492413

[28] Toh YP Karthik R Teo CC Suppiah S Cheung SL Krishna L. Toward mentoring in palliative social work: a narrative review of mentoring programs in social work. Am J Hosp Palliat Care. 2017;35:523-531. 28641444 10.1177/1049909117715216

[29] Yap HW Chua J Toh YP, et al Thematic review of mentoring in occupational therapy and physiotherapy between 2000 and 2015, sitting occupational therapy and physiotherapy in a holistic palliative medicine multidisciplinary mentoring program. J Palliat Med. 2017;2:46-55.

[30] Lee FQH Chua WJ Cheong CWS, et al A systematic scoping review of ethical issues in mentoring in surgery. J Med Educ Curric Dev. 2019;6:2382120519888915. 31903425 10.1177/2382120519888915 PMC6923696

[31] Cheong CWS Chia EWY Tay KT, et al A systematic scoping review of ethical issues in mentoring in internal medicine, family medicine and academic medicine. Adv Health Sci Educ Theory Pract. 2020;25:195-226. 31705429 10.1007/s10459-019-09934-0

[32] Singh TSS Singh A. Abusive culture in medical education: mentors must mend their ways. J Anaesth Clin Pharm. 2018;34:145-147. 10.4103/0970-9185.236659 PMC6066869 30104818

[33] Byerley JS. Mentoring in the era of# MeToo. JAMA. 2018;319:1199-1200. 29584847 10.1001/jama.2018.2128

[34] Walters KL Simoni JM Evans-Campbell TT, et al Mentoring the mentors of underrepresented racial/ethnic minorities who are conducting HIV research: beyond cultural competency. AIDS Behav. 2016;20:288-293. 27484060 10.1007/s10461-016-1491-x PMC5470932

[35] Soklaridis S Zahn C Kuper A Gillis D Taylor VH Whitehead C. Men’s fear of mentoring in the# MeToo era—what’s at stake for academic medicine? N Engl J Med. 2018;379:2270-2274. 30281387 10.1056/NEJMms1805743

[36] Olasoji HO. Broadening conceptions of medical student mistreatment during clinical teaching: message from a study of “toxic” phenomenon during bedside teaching. Adv Med Educ Pract. 2018;9:483. 29950919 10.2147/AMEP.S154642 PMC6016591

[37] Duck S Stratagems, spoils, and a serpent’s tooth: on the delights and dilemmas of personal relationships. In: Cupach WR Spitzberg BH, eds. The Dark Side of Interpersonal Communication. Hillsdale, NJ: Erlbaum, 1994:3-24.

[38] Chopra V Edelson DP Saint S. Mentorship malpractice. JAMA. 2016;315:1453-1454. 27115263 10.1001/jama.2015.18884

[39] Long J. The dark side of mentoring. Aust Educ Res. 1997;24:115-133.

[40] Walensky RP Kim Y Chang Y, et al The impact of active mentorship: results from a survey of faculty in the Department of Medicine at Massachusetts General Hospital. BMC Med Educ. 2018;18:108. 29751796 10.1186/s12909-018-1191-5 PMC5948924

[41] Krishna LKR Renganathan Y Tay KT, et al Educational roles as a continuum of mentoring’s role in medicine—a systematic review and thematic analysis of educational studies from 2000 to 2018. BMC Med Educ. 2019;19:439. 31775732 10.1186/s12909-019-1872-8 PMC6882248

[42] Arksey H O’Malley L. Scoping studies: towards a methodological framework. Int J Soc Res Methodol. 2005;8:19-32.

[43] Du Mont J Macdonald S Kosa D Elliot S Spencer C Yaffe M Development of a comprehensive hospital-based elder abuse intervention: an initial systematic scoping review. PLoS One. 2015;10:e0125105. 25938414 10.1371/journal.pone.0125105 PMC4418829

[44] O’Donovan J O’Donovan C Kuhn I Sachs SE Winters N. Ongoing training of community health workers in low-income andmiddle-income countries: a systematic scoping review of the literature. BMJ Open. 2018;8:e021467. 10.1136/bmjopen-2017-021467 PMC5931295 29705769

[45] Lim SYS Koh EYH Tan BJX Toh YP Mason S Krishna LKR Enhancing geriatric oncology training through a combination of novice mentoring and peer and near-peer mentoring: a thematic analysis of mentoring in medicine between 2000 and 2017. J Geriatr Oncol. 2020;11:566-575. 31699675 10.1016/j.jgo.2019.09.008

[46] Chong JY Ching AH Renganathan Y, et al Enhancing mentoring experiences through e-mentoring: a systematic scoping review of e-mentoring programs between 2000 and 2017. Adv Health Sci Educ Theory Pract. 2019;25:195-226. 30830505 10.1007/s10459-019-09883-8

[47] Tan YS Teo SWA Pei Y, et al A framework for mentoring of medical students: thematic analysis of mentoring programmes between 2000 and 2015. Adv Health Sci Educ Theory Pract. 2018: 23:671-697. 29550907 10.1007/s10459-018-9821-6

[48] Low CQT Toh YL Teo SWA Toh YP Krishna L A narrative review of mentoring programmes in general practice. Educ Prim Care. 2018;29:259-267. 30059278 10.1080/14739879.2018.1474723

[49] Hee JM Yap HW Ong ZX, et al Understanding the mentoring environment through thematic analysis of the learning environment in medical education: a systematic review. J Gen Intern Med. 2019;34:2190-2199. 31011975 10.1007/s11606-019-05000-y PMC6816739

[50] Sheri K Too JYJ Chuah SEL Toh YP Mason S Krishna LKR A scoping review of mentor training programs in medicine between 1990 and 2017. Med Educ Online. 2019;24:1555435. 31671284 10.1080/10872981.2018.1555435 PMC6327936

[51] Taufeeq Wahab M Bin Mohamad Ikbal MF Jingting W Wesley LTW Kanesvaran R Radha Krishna LK. Creating effective interprofessional mentoring relationships in palliative care- lessons from medicine, nursing, surgery and social work. J Palliat Care Med. 2016;6:1-10.

[52] Toh YP. Developing palliative care physicians through mentoring relationships. Palliat Med Care. 2017;4:1-6.

[53] Jia Min Hee HWY Zheng Xuan Ong Simone Quek Ying Pin Toh Stephen Mason lalit kumar radha krishna. Understanding the mentoring environment through thematic analysis of the learning environment in medical education: a systematic review. J Gen Intern Med. 2019;34:2190-2199. 31011975 10.1007/s11606-019-05000-y PMC6816739

[54] Wahab M Ikbal M Wu J Loo T Kanesvaran R Lalit K. Toward an interprofessional mentoring program in palliative care—a review of undergraduate and postgraduate mentoring in medicine, nursing, surgery and social work. J Palliat Care Med. 2016;6:1-14.

[55] Levac D Colquhoun H O’Brien KK. Scoping studies: advancing the methodology. Implement Sci. 2010;5:69. 20854677 10.1186/1748-5908-5-69 PMC2954944

[56] Chambers D Wilson P Thompson C Harden M. Social network analysis in healthcare settings: a systematic scoping review. PLoS One. 2012;7:e41911. 22870261 10.1371/journal.pone.0041911 PMC3411695

[57] Colquhoun HL Levac D O’Brien KK, et al Scoping reviews: time for clarity in definition, methods, and reporting. J Clin Epidemiol. 2014;67:1291-1294. 25034198 10.1016/j.jclinepi.2014.03.013

[58] Thomas A Menon A Boruff J Rodriguez AM Ahmed S. Applications of social constructivist learning theories in knowledge translation for healthcare professionals: a scoping review. Implement Sci. 2014;9:54. 24885925 10.1186/1748-5908-9-54 PMC4040365

[59] Mays N Roberts E Popay J. Synthesising research evidence. In: Allen P Black N Clarke A Fulop N Anderson S (eds) Studying the organisation and delivery of health services: Research methods. London: Routledge; 2001:240.

[60] Lorenzetti DL Powelson SE. A scoping review of mentoring programs for academic librarians. J. Acad. Librariansh. 2015;41(2):186-196.

[61] Osama T Brindley D Majeed A, et al Teaching the relationship between health and climate change: a systematic scoping review protocol. BMJ Open. 2018;8(5):e020330. 10.1136/bmjopen-2017-020330 PMC5961595 29780026

[62] Physicians ACo. Subspecialties of internal medicine. Philadelphia, PA: American College of Physicians. Published 2018 Accessed May 20, 2018.

[63] Peters M Godfrey C McInerney P Soares C. Khalil H Parker D The Joanna Briggs Institute reviewers’ manual 2015: methodology for JBI scoping reviews. 2015 http://joannabriggs.org/assets/docs/sumari/Reviewers-Manual_Methodology-for-JBI-Scoping-Reviews_2015_v1.pdf. Accessed April 29, 2019.

[64] Peters MD Godfrey CM Khalil H McInerney P Parker D Soares CB. Guidance for conducting systematic scoping reviews. Int J Evid Based Healthc. 2015;13(3):141-146. 26134548 10.1097/XEB.0000000000000050

[65] Krishna L Toh Y Mason S Kanesvaran R. Mentoring stages: a study of undergraduate mentoring in palliative medicine in Singapore. PLoS one. 2019;14(4):e0214643-e0214643. 31017941 10.1371/journal.pone.0214643 PMC6481808

[66] Ikbal MFBM Wu JT Wahab MT Kanesvaran R Krishna LKR Mentoring in palliative medicine: guiding program design through thematic analysis of mentoring in internal medicine between 2000 and 2015. J Palliat Care Med. 2017;7:318.

[67] Sambunjak D Straus SE Marusic A. A systematic review of qualitative research on the meaning and characteristics of mentoring in academic medicine. J Gen Intern Med. 2010;25:72-78. 10.1007/s11606-009-1165-8 PMC2811592 19924490

[68] Braun V Clarke V. Using thematic analysis in psychology. Qualitative Research in Psychology. 2006;3:77-101.

[69] Wen SH Ren WM Qu L Wang Y Carline JD Fang GE. A survey on financial support and research achievement of medical education research units in China. Med Teach. 2011;33:e158-e162. 21345055 10.3109/0142159X.2010.543442

[70] Varpio L Gruppen L Hu W, et al Working definitions of the roles and an organizational structure in health professions education scholarship: initiating an international conversation. Acad Med 2017;92:205-208. 27580432 10.1097/ACM.0000000000001367

[71] Davis MH Karunathilake I Harden RM. AMEE Education Guide no. 28: the development and role of departments of medical education. Med Teach. 2005;27:665-675. 16451885 10.1080/01421590500398788

[72] Hsieh H-F Shannon SE. Three approaches to qualitative content analysis. Quale Health Res. 2005;15:1277-1288. 10.1177/1049732305276687 16204405

[73] Neal JW Neal ZP Lawlor JA Mills KJ McAlindon K. What makes research useful for public school educators? Adm Policy Ment Health. 2018;45:432-446. 29124526 10.1007/s10488-017-0834-x PMC5878984

[74] Soemantri D Herrera C Riquelme A. Measuring the educational environment in health professions studies: a systematic review. Med Teach. 2010;32:947-952. 21090946 10.3109/01421591003686229

[75] Schönrock-Adema J Heijne-Penninga M van Hell EA Cohen-Schotanus J. Necessary steps in factor analysis: enhancing validation studies of educational instruments. The PHEEM applied to clerks as an example. Med Teach. 2009;31:e226-e232. 19089728 10.1080/01421590802516756

[76] Riquelme A Herrera C Aranis C Oporto J Padilla O. Psychometric analyses and internal consistency of the PHEEM questionnaire to measure the clinical learning environment in the clerkship of a Medical School in Chile. Med Teach. 2009;31:e221-e225. 19811154 10.1080/01421590902866226

[77] Gordon M Gibbs T. STORIES statement: publication standards for healthcare education evidence synthesis. BMC Med. 2014;12:143. 25190085 10.1186/s12916-014-0143-0 PMC4243720

[78] Vaismoradi M Turunen H Bondas T. Content analysis and thematic analysis: Implications for conducting a qualitative descriptive study. Nurs Health Sci. 2013;15:398-405. 23480423 10.1111/nhs.12048

[79] Moher D Liberati A Tetzlaff J Altman DG. Preferred reporting items for systematic reviews and meta-analyses: the PRISMA statement. Ann Intern Med. 2009;151:264-269. 19622511 10.7326/0003-4819-151-4-200908180-00135

[80] Reed DA Beckman TJ Wright SM Levine RB Kern DE Cook DA. Predictive validity evidence for medical education research study quality instrument scores: quality of submissions to JGIM’s Medical Education Special Issue. J Gen Intern Med. 2008;23:903-907. 18612715 10.1007/s11606-008-0664-3 PMC2517948

[81] Tong A Sainsbury P Craig J. Consolidated criteria for reporting qualitative research (COREQ): a 32-item checklist for interviews and focus groups. Int J Qual Health Care. 2007;19:349-357. 17872937 10.1093/intqhc/mzm042

[82] Haig A Dozier M. BEME Guide no 3: systematic searching for evidence in medical education–Part 1: Sources of information. Med Teach. 2003;25:352-363. 12893544 10.1080/0142159031000136815

[83] Frei E Stamm M Buddeberg-Fischer B. Mentoring programs for medical students-a review of the PubMed literature 2000-2008. BMC Med Educ. 2010;10:32. 20433727 10.1186/1472-6920-10-32 PMC2881011

[84] White HK Buhr GT Pinheiro SO. Mentoring: a key strategy to prepare the next generation of physicians to care for an aging America. J Am Geriatr Soc. 2009;57:1270-1277. 19582901 10.1111/j.1532-5415.2009.02300.x

[85] Farkas AH Allenbaugh J Bonifacino E Turner R Corbelli JA. Mentorship of US medical students: a systematic review. J Gen Intern Med. 2019;34:2602-2609. 31485967 10.1007/s11606-019-05256-4 PMC6848625

[86] Lin C-D Lin BY-J Lin C-C Lee C-C. Redesigning a clinical mentoring program for improved outcomes in the clinical training of clerks. Med Educ Online. 2015;20:28327. 26384479 10.3402/meo.v20.28327 PMC4575418

[87] Devi V Abraham RR Adiga A Ramnarayan K Kamath A. Fostering research skills in undergraduate medical students through Mentored Student Projects: example from an Indian medical school. Kathmandu University Med J. 2010;8:294-298. 10.3126/kumj.v8i3.6215 22610733

[88] Dobie S Smith S Robins L. How assigned faculty mentors view their mentoring relationships: an interview study of mentors in medical education. Mentor Tutor. 2010;18:337-359.

[89] Fleming M House MS Shewakramani MV, et al The mentoring competency assessment: validation of a new instrument to evaluate skills of research mentors. Acad Med 2013;88:1002. 23702534 10.1097/ACM.0b013e318295e298 PMC3727250

[90] Straus SE Johnson MO Marquez C Feldman MD. Characteristics of successful and failed mentoring relationships: a qualitative study across two academic health centers. Acad Med. 2013;88:82. 23165266 10.1097/ACM.0b013e31827647a0 PMC3665769

[91] Fraser A. Mentoring resident doctors. N Zealand Med J. 2004;117:1-5. 15505670

[92] Ikbal M Wu J Wahab M Kanesvaran R Krishna L. Mentoring in palliative medicine: Guiding program design through thematic analysis of mentoring in internal medicine between 2000 and 2015. J Palliat Care Med. 2017;7:318.

[93] Mark S Link H Morahan PS Pololi L Reznik V Tropez-Sims S. Innovative mentoring programs to promote gender equity in academic medicine. Acad Med. 2001;76:39-42. 11154192 10.1097/00001888-200101000-00011

[94] Gotterer GS O’day D Miller BM. The Emphasis program: a scholarly concentrations program at Vanderbilt University School of Medicine. Acad Med. 2010;85:1717-1724. 20671539 10.1097/ACM.0b013e3181e7771b

[95] Usmani A Omaeer Q Sultan ST. Mentoring undergraduate medical students: experience from Bahria University Karachi. J Pak Med Assoc. 2011;61:790. 22356004

[96] Shamim MS. Mentoring programme for faculty in medical education: South-Asian perspective. J Pak Med Assoc. 2013;63:619-623. 23757992

[97] Sheikh ASF Sheikh SA Huynh M-H Mohamed MA Mentoring among Pakistani postgraduate resident doctors. Postgraduate Med J. 2017;93:115-120. 10.1136/postgradmedj-2016-134060 27343051

[98] Kalén S Ponzer S Seeberger A Kiessling A Silén C. Longitudinal mentorship to support the development of medical students’ future professional role: a qualitative study. BMC Med Educ. 2015;15:97. 26037407 10.1186/s12909-015-0383-5 PMC4458053

[99] Dzau VJ Soo KC Mentorship in academic medicine: a catalyst of talents. Ann Acad Med Singapore. 2015;44:232-234. 26377056

[100] Winston KA Van Der Vleuten CP Scherpbier AJ. The role of the teacher in remediating at-risk medical students. Med Teach. 2012;34:e732-e742. 22658068 10.3109/0142159X.2012.689447

[101] Sng JH Pei Y Toh YP Peh TY Neo SH Krishna LKR Mentoring relationships between senior physicians and junior doctors and/or medical students: a thematic review. Med Teach. 2017;39:866-875. 28562193 10.1080/0142159X.2017.1332360

[102] Kashiwagi DT Varkey P Cook DA. Mentoring programs for physicians in academic medicine: a systematic review. Acad Med. 2013;88:1029-1037. 23702518 10.1097/ACM.0b013e318294f368

[103] Boninger M Troen P Green E, et al Implementation of a longitudinal mentored scholarly project: an approach at two medical schools. Acad Med. 2010;85:429-437. 20182115 10.1097/ACM.0b013e3181ccc96f

[104] Coates WC Crooks K Slavin SJ Guiton G Wilkerson L. Medical school curricular reform: fourth-year colleges improve access to career mentoring and overall satisfaction. Acad Med. 2008;83:754-760. 18667890 10.1097/ACM.0b013e31817eb7dc

[105] Fornari A Murray TS Menzin AW, et al Mentoring program design and implementation in new medical schools. Med Educ Online. 2014;19:24570. 24962112 10.3402/meo.v19.24570 PMC4069409

[106] von der Borch P Dimitriadis K Störmann S, et al A novel large-scale mentoring program for medical students based on a quantitative and qualitative needs analysis. GMS Zeitschrift für medizinische Ausbildung. 2011;28:1-16. 10.3205/zma000738 PMC3149462 21818236

[107] Davis OC Nakamura J. A proposed model for an optimal mentoring environment for medical residents: a literature review. Acad Med. 2010;85:1060-1066. 20505410 10.1097/ACM.0b013e3181dc4aab

[108] Toklu HZ Fuller JC. Mentor-mentee relationship: a win-win contract in graduate medical education. Cureus. 2017;9:e1908. 29435394 10.7759/cureus.1908 PMC5798810

[109] Schäfer M Pander T Pinilla S Fischer MR von der Borch P Dimitriadis K. The Munich-Evaluation-of-Mentoring-Questionnaire (MEMeQ)–a novel instrument for evaluating protégés’ satisfaction with mentoring relationships in medical education. BMC Med Educ. 2015;15:201. 26553241 10.1186/s12909-015-0469-0 PMC4640154

[110] Harrison R Anderson J Laloë P-A Santillo M Lawton R Wright J. Mentorship for newly appointed consultants: what makes it work? Postgrad Med J. 2014;90:439-445. 24951513 10.1136/postgradmedj-2013-132333

[111] Pinilla S Pander T von der Borch P Fischer MR Dimitriadis K. 5 years of experience with a large-scale mentoring program for medical students. GMS Zeitschrift für Medizinische Ausbildung. 2015;32. 10.3205/zma000947 PMC4330635 25699108

[112] Buddeberg-Fischer B Herta K-D. Formal mentoring programmes for medical students and doctors–a review of the Medline literature. Med Teach. 2006;28:248-257. 16753724 10.1080/01421590500313043

[113] DeFilippis E Cowell E Rufin M Sansone S Kang Y. Innovative mentoring for female medical students. Clin Teach. 2016;13:381-382. 26084472 10.1111/tct.12408

[114] Hauer KE Teherani A Dechet A Aagaard EM. Medical students’ perceptions of mentoring: a focus-group analysis. Med Teach. 2005;27:732-734. 16451896 10.1080/01421590500271316

[115] Beech BM Calles-Escandon J Hairston KG Langdon MSE Latham-Sadler BA Bell RA. Mentoring programs for underrepresented minority faculty in academic medical centers: a systematic review of the literature. Acad Med 2013;88. 10.1097/ACM.0b013e31828589e3 PMC3835658 23425989

[116] Bhatia A Singh N Dhaliwal U. Mentoring for first year medical students: humanising medical education. Indian J Med Ethics. 2013;10:100-103. 23697488 10.20529/IJME.2013.030

[117] Han E-R Chung E-K Oh S-A Woo Y-J Hitchcock MA. Mentoring experience and its effects on medical interns. Singapore Med J. 2014;55:593. 25631971 10.11622/smedj.2014157 PMC4294009

[118] Iversen AC Eady NA Wessely SC. The role of mentoring in academic career progression: a cross-sectional survey of the Academy of Medical Sciences mentoring scheme. Journal of the Royal Society of Medicine. 2014;107(8):308-317. 24739382 10.1177/0141076814530685 PMC4128076

[119] Kalén S Ponzer S Silén C. The core of mentorship: medical students’ experiences of one-to-one mentoring in a clinical environment. Adv Health Sci Educ. 2012;17:389-401. 10.1007/s10459-011-9317-0 21792708

[120] Arnold L Cuddy PG Hathaway SB Quaintance JL Kanter SL. Medical school factors that prepare students to become leaders in medicine. Acad Med. 2018;93:274-282. 28991842 10.1097/ACM.0000000000001937

[121] Ottenheijm RP Zwietering PJ Scherpbier AJ Metsemakers JF. Early student-patient contacts in general practice: an approach based on educational principles. Med Teach. 2008;30:802-808. 18608956 10.1080/01421590802047265

[122] Thomas-Squance GR Goldstone R Martinez A Flowers LK. Mentoring of students from under-represented groups using emotionally competent processes and content. Med Educ. 2011;45:1153-1154. 21933254 10.1111/j.1365-2923.2011.04133.x

[123] Schmidt A Schwedler A Hahn EG. Does the training of mentors increase the contact frequency and the quality of support in a portfolio-based teaching module? GMS Z Med Ausbild. 2010;27:1-10. 10.3205/zma000706 PMC3140371 21818214

[124] Meinel FG Dimitriadis K von der Borch P Störmann S Niedermaier S Fischer MR. More mentoring needed? A cross-sectional study of mentoring programs for medical students in Germany. BMC Med Educ. 2011;11:68. 21943281 10.1186/1472-6920-11-68 PMC3191506

[125] Ludwig B Turk B Seitz T Klaus I Löffler-Stastka H. The search for attitude—a hidden curriculum assessment from a central European perspective. Wien Klin Wochenschr. 2018;130:134-140. 29356896 10.1007/s00508-018-1312-5 PMC5816099

[126] Thomas-MacLean R Hamoline R Quinlan E Ramsden VR Kuzmicz J. Discussing mentorship: an ongoing study for the development of a mentorship program in Saskatchewan. Can Fam Physician. 2010;56:e263-e272. 20631262 PMC2922829

[127] Srinivasan M Li S-TT Meyers FJ, et al “Teaching as a competency”: competencies for medical educators. Acad Med. 2011;86:1211-1220. 21869655 10.1097/ACM.0b013e31822c5b9a

[128] Longo DR Katerndahl DA Turban DB, et al The research mentoring relationship in family medicine: findings from the grant generating project. Fam Med-Kans City. 2011;43:240. 21499996

[129] Straus SE Chatur F Taylor M. Issues in the mentor–mentee relationship in academic medicine: a qualitative study. Acad Med. 2009;84:135-139. 19116493 10.1097/ACM.0b013e31819301ab

[130] Stenfors-Hayes T Kalén S Hult H Dahlgren LO Hindbeck H Ponzer S. Being a mentor for undergraduate medical students enhances personal and professional development. Med Teach. 2010;32:148-153. 20163231 10.3109/01421590903196995

[131] Sozio SM Chan KS Beach MC. Development and validation of the Medical Student Scholar-Ideal Mentor Scale (MSS-IMS). BMC Med Educ. 2017;17:132. 28789660 10.1186/s12909-017-0969-1 PMC5549328

[132] Sanfey H Hollands C Gantt NL. Strategies for building an effective mentoring relationship. Am J Surg. 2013;206:714-718. 24157350 10.1016/j.amjsurg.2013.08.001

[133] Zier K Coplit LD. Introducing INSPIRE, a scholarly component in undergraduate medical education. Mount Sinai J Med. 2009;76:387-391. 10.1002/msj.20121 19642153

[134] Levy BD Katz JT Wolf MA Sillman JS Handin RI Dzau VJ. An initiative in mentoring to promote residents’ and faculty members’ careers. Acad Med. 2004;79:845-850. 15326007 10.1097/00001888-200409000-00006

[135] Stamm M Buddeberg-Fischer B. The impact of mentoring during postgraduate training on doctors’ career success. Med Educ. 2011;45:488-496. 21486324 10.1111/j.1365-2923.2010.03857.x

[136] Miedzinski LJ Wong WW Morrison JC. Perceptions of a faculty mentorship programme. Med Educ. 2009;43:1084-1084. 10.1111/j.1365-2923.2009.03468.x 19799724

[137] Guraya SY Guraya SS Almaramhy HH. The legacy of teaching medical professionalism for promoting professional practice: a systematic review. Biomed Pharmacol J. 2016;9:809-817.

[138] Morrison LJ Lorens E Bandiera G, et al Impact of a formal mentoring program on academic promotion of Department of Medicine faculty: a comparative study. Med Teach. 2014;36:608-614. 24804918 10.3109/0142159X.2014.899683

[139] Rothberg MB Kleppel R Friderici JL Hinchey K. Implementing a resident research program to overcome barriers to resident research. Acad Med. 2014;89:1133-1139. 24751975 10.1097/ACM.0000000000000281

[140] Luckhaupt SE Chin MH Mangione CM, et al Mentorship in academic general internal medicine. J Gen Intern Med. 2005;20:1014-1018. 16307626 10.1111/j.1525-1497.2005.215.x PMC1350921

[141] Larkin GL. Mapping, modeling, and mentoring: charting a course for professionalism in graduate medical education. Camb Q Healthc Ethics. 2003;12:167-177. 12764882 10.1017/s0963180103122062

[142] Elez E Quintanar T Bosch-Barrera J, et al The medical oncology resident mentor: situation and workload. Clin Transl Oncol. 2019;21:304-313. 30062521 10.1007/s12094-018-1923-3

[143] Manabe YC Nambooze H Okello ES, et al Group mentorship model to enhance the efficiency and productivity of PhD research training in Sub-Saharan Africa. Ann Glob Health. 2018;84:170-175. 30873808 10.29024/aogh.25 PMC6748251

[144] Spence JP Buddenbaum JL Bice PJ Welch JL Carroll AE. Independent investigator incubator (I3): a comprehensive mentorship program to jumpstart productive research careers for junior faculty. BMC Med Educ. 2018;18:186. 30081899 10.1186/s12909-018-1290-3 PMC6080403

[145] Sambunjak D Straus SE Marušić A. Mentoring in academic medicine: a systematic review. JAMA. 2006;296:1103-1115. 16954490 10.1001/jama.296.9.1103

[146] Ramani S. Twelve tips to promote excellence in medical teaching. Med Teach. 2006;28:19-23. 16627316 10.1080/01421590500441786

[147] Dimitriadis K von der Borch P Störmann S, et al Characteristics of mentoring relationships formed by medical students and faculty. Med Educ. 2012;17:17242. 10.3402/meo.v17i0.17242 PMC3443398 22989620

[148] Kukreja S Chhabra N Kaur A Arora R Singh T. Introducing mentoring to 1st-year medical students of a private medical college in North India: A pilot study. Int J Appl Basic Med Res. 2017;7(Suppl 1):S67. 29344462 10.4103/ijabmr.IJABMR_160_17 PMC5769175

[149] Hawkins A Jones K Stanton A A mentorship programme for final-year students. Clin Teach. 2014;11:345-349. 25041666 10.1111/tct.12149

[150] Kalén S Stenfors-Hayes T Hylin U Larm MF Hindbeck H Ponzer S Mentoring medical students during clinical courses: a way to enhance professional development. Med Teach. 2010;32:e315-e321. 10.3109/01421591003695295 20662566

[151] Zuzuárregui JRP Hohler AD Comprehensive Opportunities for Research and Teaching Experience (CORTEX): a mentorship program. Neurology. 2015;84:2372-2376. 25957332 10.1212/WNL.0000000000001663

[152] Goldszmidt MA Zibrowski EM Watling CJ Fostering education scholarship: the mentored research group. Med Educ. 2009;43:1084-1085. 10.1111/j.1365-2923.2009.03497.x 19874505

[153] Kwan JY Prokubovskaya A Hopman WM Carpenter J Mentoring for female medical trainees in a dual-setting group. Med Educ. 2015;49:540-540. 10.1111/medu.12682 25924161

[154] Sakushima K Mishina H Fukuhara S, et al Mentoring the next generation of physician-scientists in Japan: a cross-sectional survey of mentees in six academic medical centers. BMC Med Educ. 2015;15:54. 25890341 10.1186/s12909-015-0333-2 PMC4373037

[155] Ho Y Kwon OY Park SY Yoon TY A study of satisfaction of medical students on their mentoring programs at one medical school in Korea. Korean J Med Educ. 2017;29:253. 29207456 10.3946/kjme.2017.71 PMC5717413

[156] Sayan M Ohri N Lee A, et al The Impact of Formal Mentorship Programs on Mentorship Experience Among Radiation Oncology Residents From the Northeast. Front Oncol. 2019;9:1369. 31867278 10.3389/fonc.2019.01369 PMC6904328

[157] Pham MT Rajić A Greig JD Sargeant JM Papadopoulos A McEwen SA. A scoping review of scoping reviews: advancing the approach and enhancing the consistency. Res Synth Methods. 2014;5:371-385. 26052958 10.1002/jrsm.1123 PMC4491356

