# Supplementary material for: The Pivotal Role of Host Organizations in Enhancing Mentoring in Internal Medicine: A Scoping Review
Source: J Med Educ Curric Dev. 2020 Sep 30;7:2382120520956647. doi: 10.1177/2382120520956647 (PMC7536487; doi:10.1177/2382120520956647)
Supplement: Supplementary_material – Supplemental material for The Pivotal Role of Host Organizations in Enhancing Mentoring in Internal Medicine: A Scoping Review [file Supplementary_material.pdf]

Appendices

Appendix A – PubMed Search Strategy

(mentoring[MeSH] OR mentors[MeSH] OR mentor[tiab] OR mentors[tiab] OR mentoring[tiab] OR mentorship[tiab] OR "mentoring structure"[tiab] OR "mentoring structures"[tiab] OR "mentoring relationship"[tiab] OR "mentoring relationships"[tiab] OR "mentoring environment"[tiab] OR "mentoring environments"[tiab]) AND ("Schools, Medical"[MeSH] OR "Medicine"[MeSH] OR "Students, Medical"[MeSH] OR "medical student"[tiab] OR "medical students"[tiab] OR "medical school"[tiab] OR "medical schools"[tiab] OR medicine[tiab])

Appendix B – Characteristics of Included Articles

Types of Papers

Type of Paper	Number	Papers
Qualitative	12	Arnold et al. 2017<br>Bhatia et al. 2013<br>Dobie et al. 2010<br>Harrison et al. 2014<br>Hauer et al. 2005<br>Jackson et al. 2003<br>Kalén et al. 2015<br>Kalén et al. 2012<br>Stenfors-Hayes et al. 2010<br>Straus et al. 2009<br>Straus et al. 2013<br>Thomas-MacLean et al. 2011
Quantitative	22	Spence et al. 2018<br>Manabe et al. 2018<br>DeFilippis et al. 2016<br>Devi et al. 2010<br>Fleming et al. 2013<br>Goldszmidt et al. 2009<br>Han et. al 2014<br>Kwan et al. 2015<br>Lin et al. 2015<br>Longo et al. 2011<br>Luckhaupt et al. 2005<br>Ludwig et al. 2018<br>Miedzinski et al. 2009<br>Morrison et al. 2014<br>Ramanan et al. 2006<br>Sakushima et al. 2015<br>Schmidt et al. 2010<br>Sheikh et al. 2017<br>Sozio et al. 2017<br>Stamm et al. 2011<br>Sayan et al. 2019<br>Elez et al. 2018
Mixed qualitative and quantitative	13	Coates et al. 2008<br>Dimitriadis et al. 2012<br>Fornari et al. 2014<br>Hawkins et al. 2014<br>Ho et al. 2017<br>Iversen et al. 2014<br>Kalén et al. 2010<br>Kukreja et al. 2017<br>Meinel et al. 2011<br>Schafer et al. 2015<br>Usmani et al. 2011<br>von der Borch et al. 2011<br>Winston et al. 2012
Descriptive	5	Larkin et al. 2003<br>Mark et al. 2001<br>Sanfey et al. 2013<br>Shamim et al. 2013<br>Srinivasan et al. 2011
Report	8	Boninger et al. 2010<br>Gotterer et al. 2010

		Levy et al. 2004<br>Pinilla et al. 2015<br>Rothberg et al. 2014<br>Thomas-Squance et al. 2011<br>Zier et al. 2009<br>Zuzuarregui et al. 2015
Editorial	2	Dzau et al. 2015<br>Toklu et al. 2017
Perspective	1	Fraser et al. 2004
Systematic Review	10	Beech et al. 2013<br>Frei et al. 2010<br>Guraya et al. 2016<br>Ikbal et al. 2017<br>Kashiwagi et al. 2013<br>Sambunjak et al. 2010<br>Sambunjak et al. 2010<br>Sng et al. 2017<br>Tan et al. 2018<br>Farkas et al. 2019
Literature Review	3	Buddeberg-Fischer et al. 2006<br>Davis et al. 2010<br>Ottenheijm et al. 2008

**Time Point of Paper**	**Number**	**Papers**
Retrospective	42	Arnold et al. 2017<br>Bhatia et al. 2013<br>DeFilippis et al. 2016<br>Devi et al. 2010<br>Dobie et al. 2010<br>Fornari et al. 2014<br>Goldszmidt et al. 2009<br>Han et. al 2014<br>Harrison et al. 2014<br>Hauer et al. 2005<br>Hawkins et al. 2014<br>Ho et al. 2017<br>Iversen et al. 2014<br>Jackson et al. 2003<br>Kalén et al. 2015<br>Kalén et al. 2012<br>Kalén et al. 2010<br>Kukreja et al. 2017<br>Kwan et al. 2015<br>Longo et al. 2011<br>Luckhaupt et al. 2005<br>Ludwig et al. 2018<br>Meinel et al. 2011<br>Miedzinski et al. 2009<br>Morrison et al. 2014<br>Ramanan et al. 2006<br>Sakushima et al. 2015<br>Schafer et al. 2015<br>Schmidt et al. 2010<br>Sheikh et al. 2017<br>Sozio et al. 2017<br>Stenfors-Hayes et al. 2010<br>Straus et al. 2009<br>Straus et al. 2013

		Thomas-MacLean et al. 2011<br>Usmani et al. 2011<br>von der Borch et al. 2011<br>Winston et al. 2012<br>Tan et al. 2018<br>Spence et al. 2018<br>Manabe et al. 2018<br>Farkas et al. 2019
Prospective	7	Coates et al. 2008<br>Dimitriadis et al. 2012<br>Fleming et al. 2013<br>Lin et al. 2015<br>Stamm et al. 2011<br>Sayan et al. 2019<br>Elez et al. 2018

Who were interviewed/ surveyed?

Addressed	Number	Papers
Mentors	6	Dobie et al. 2010<br>Jackson et al. 2003<br>Luckhaupt et al. 2005<br>Stenfors-Hayes et al. 2010<br>Usmani et al. 2011<br>Elez et al. 2018
Mentees	34	Arnold et al. 2017<br>Boninger et al. 2010<br>Coates et al. 2008<br>DeFilippis et al. 2016<br>Devi et al. 2010<br>Dimitriadis et al. 2012<br>Goldszmidt et al. 2009<br>Gotterer et al. 2010<br>Han et. al 2014<br>Harrison et al. 2014<br>Hauer et al. 2005<br>Ho et al. 2017<br>Kalén et al. 2015<br>Kalén et al. 2012<br>Kalén et al. 2010<br>Kwan et al. 2015<br>Levy et al. 2004<br>Lin et al. 2015<br>Ludwig et al. 2018<br>Morrison et al. 2014<br>Pinilla et al. 2015<br>Ramanan et al. 2006<br>Rothberg et al. 2014<br>Sakushima et al. 2015<br>Schafer et al. 2015<br>Schmidt et al. 2010<br>Sozio et al. 2017<br>Stamm et al. 2011<br>Thomas-Squance et al. 2011<br>Zier et al. 2009<br>Zuzuarregui et al. 2015<br>Manabe et al. 2018<br>Spence et al. 2018

		Sayan et al. 2019
Both mentees and mentors	12	Bhatia et al. 2013<br>Fleming et al. 2013<br>Hawkins et al. 2014<br>Kukreja et al. 2017<br>Longo et al. 2011<br>Miedzinski et al. 2009<br>Sheikh et al. 2017<br>Straus et al. 2009<br>Straus et al. 2013<br>Thomas-MacLean et al. 2011<br>von der Borch et al. 2011<br>Winston et al. 2012
Host organization	2	Fornari et al. 2014<br>Meinel et al. 2011
Mentees, mentors and host organization	1	Iversen et al. 2014
